# Effects of Obesity and Metabolic Syndrome on Steroidogenesis and Folliculogenesis in the Female Ossabaw Mini-Pig

**DOI:** 10.1371/journal.pone.0128749

**Published:** 2015-06-05

**Authors:** Annie E. Newell-Fugate, Jessica N. Taibl, Mouhamad Alloosh, Michael Sturek, Janice M. Bahr, Romana A. Nowak, Rebecca L. Krisher

**Affiliations:** 1 Department of Animal Sciences, University of Illinois at Urbana-Champaign, Urbana, IL 61801, United States of America; 2 Department of Cellular & Integrative Physiology, Indiana University School of Medicine, Indianapolis, Indiana 46202–5120, United States of America; INIA, SPAIN

## Abstract

The discrete effects of obesity on infertility in females remain undefined to date. To investigate obesity-induced ovarian dysfunction, we characterized metabolic parameters, steroidogenesis, and folliculogenesis in obese and lean female Ossabaw mini-pigs. Nineteen nulliparous, sexually mature female Ossabaw pigs were fed a high fat/cholesterol/fructose diet (n=10) or a control diet (n=9) for eight months. After a three-month diet-induction period, pigs remained on their respective diets and had ovarian ultrasound and blood collection conducted during a five-month study period after which ovaries were collected for histology, cell culture, and gene transcript level analysis. Blood was assayed for steroid and protein hormones. Obese pigs developed abdominal obesity and metabolic syndrome, including hyperglycemia, hypertension, insulin resistance and dyslipidemia. Obese pigs had elongated estrous cycles and hyperandrogenemia with decreased LH, increased FSH and luteal phase progesterone, and increased numbers of medium, ovulatory, and cystic follicles. Theca cells of obese, compared to control, pigs displayed androstenedione hypersecretion in response to in vitro treatment with LH, and up-regulated 3-beta-hydroxysteroid dehydrogenase 1 and 17-beta-hydroxysteroid dehydrogenase 4 transcript levels in response to in vitro treatment with LH or LH + insulin. Granulosa cells of obese pigs had increased 3-beta-hydroxysteroid dehydrogenase 1 transcript levels. In summary, obese Ossabaw pigs have increased transcript levels and function of ovarian enzymes in the delta 4 steroidogenic pathway. Alterations in LH, FSH, and progesterone, coupled with theca cell dysfunction, contribute to the hyperandrogenemia and disrupted folliculogenesis patterns observed in obese pigs. The obese Ossabaw mini-pig is a useful animal model in which to study the effects of obesity and metabolic syndrome on ovarian function and steroidogenesis. Ultimately, this animal model may be useful toward the development of therapies to improve fertility in obese and/or hyperandrogenemic females or in which to examine the effects of obesity on the maternal-fetal environment and offspring health.

## Introduction

Two thirds of Americans are overweight or obese [1], rendering the obesity epidemic a challenging public health problem for the United States health care system. Obesity, particularly abdominal obesity, is intimately associated with the development of metabolic syndrome (MetS) [2] and its sequelae of cardiovascular disease and type 2 diabetes. One of the long-term health implications associated with obesity is the adverse effect on female fertility, resulting in significant ovulatory dysfunction and oligomenorrhea [3], an increased rate of miscarriage [4], and increased gestational complications [5]. Furthermore, certain reproductive disorders like polycystic ovary syndrome may be exacerbated by obesity. With respect to the endocrine milieu, oligo/anovulatory obese women are hyperandrogenemic with decreased serum LH levels, may be normo or hyperestrogenemic,[6], have low serum progesterone, and increased sensitivity of the pituitary to gonadotropins [7]. Improvement of reproductive function in obese women necessitates multimodal therapy targeted at adipose tissue, the reproductive tract, and the endocrine system. Treatments for obesity-induced infertility include: weight loss through a low energy diet, exercise, and/or surgical means [8, 9]; use of insulin sensitizing agents as necessary[10]; and assisted reproductive techniques like in vitro fertilization (IVF) and intracytoplasmic sperm injection [11, 12].

Multiple animal models have been used to study obesity [13] and obesity-associated ovarian dysfunction [14]. However, many of these models are genetically modified strains of rodents fed high fat diets and may not mimic the pathogenesis of obesity in humans. Furthermore, the ability to follow longitudinal changes in biochemical parameters over time is limited in rodent models. Although non-human primate models of obesity closely model the disease manifestation in humans, the cost of primates, the long lag time required for them to reach adulthood, as well as the increased zoonotic disease risk in these species render them inaccessible to large scale research [15]. Therefore, the development of a large animal model that addresses the effects of obesity on ovarian function would have a significant positive impact on obesity research. Pigs are a valuable tool for the study of metabolic diseases in humans due to their similar lipoprotein profiles, adipose tissue composition (white vs. brown), and similar gastrointestinal tract anatomy and physiology [16]. Additionally, there are similarities between cycle length, LH receptor location and function, and length of time between the LH surge and ovulation in humans and pigs [17]. With these similarities in mind, our goal was to examine the Ossabaw miniature pig as an animal model for the effects of obesity on ovarian function in women. Ossabaw pigs have a loss of function mutation in the Val199→IIe region of the PRKAG3 gene (the γ3 isoform of AMP-activated protein kinase) that is associated with increased intramuscular fat and is consistent with the obese, “thrifty” genotype [18, 19]. AMPK is a metabolic “master switch” that, when stimulated, results in hepatic fatty acid oxidation and ketogenesis, inhibition of cholesterol synthesis, lipogenesis and triglyceride synthesis, inhibition of adipocyte lipolysis and lipogenesis, stimulation of skeletal muscle fatty acid oxidation and muscle glucose uptake and modulation of insulin secretion by pancreatic beta-cells.

The Ossabaw mini-pig, when fed a hypercaloric, high fat/cholesterol/fructose diet, naturally develops features of MetS including visceral obesity, glucose intolerance, insulin resistance, dyslipidemia, and hypertension [18–22]. Ossabaw pigs have the highest total body lipid of any mammal assessed to date [23, 24] and develop metabolic syndrome more rapidly and robustly than any other currently available swine model [25]. Although weight loss through diet, exercise, and behavioral modification has been shown to improve fertility outcomes in obese women [8], there is a need for an appropriate animal model in which to test novel pharmaceuticals targeted to further bolster ovarian follicle health and ovulatory function in obese patients.

We have previously characterized the reproductive effects of a hypercaloric, high fat/cholesterol/fructose diet fed long-term (13 months) to multiparous, adult female Ossabaw mini-pigs [26]. Shorter-term feeding of a hypercaloric, high fat/cholesterol/fructose diet to young pigs would minimize the cost while potentially maintaining the useful features of this animal model. This study builds upon our previous work through the examination of ovarian steroidogenic enzyme transcript levels and function in obese and control Ossabaw mini-pigs. The objectives of this study were to: 1) characterize metabolic parameters, folliculogenesis, and serum reproductive hormone concentrations in young (6 months old at diet onset), female Ossabaw mini-pigs fed either a hypercaloric, high fat/cholesterol/fructose diet or a control diet for eight months; 2) evaluate gene transcript levels of 3-beta-hydroxysteroid dehydrogenase, 17-beta-hydroxysteroid dehydrogenase, and 17α-hydroxylase/17,20 lyase/17,20 desmolase in the theca interna and 3-beta-hydroxysteroid dehydrogenase and aromatase in granulosa cells of obese and control female Ossabaw mini-pigs; 3) evaluate androgen secretion from in vitro cultured theca interna cells from obese and control female Ossabaw mini-pigs.

## Materials and Methods

All experimental procedures were performed in compliance with University of Illinois Urbana-Champaign (UIUC) Institutional Animal Care and Use Committee (IACUC) regulation and followed the guidelines of the *Guide for the Care and Use of Laboratory Animals* [27]. The experiments conducted herein were approved by the UIUC IACUC (IACUC Number: 08191).

### Husbandry and diet treatment

The 19 nulliparous Ossabaw miniature pigs (*Sus scrofa)* used in this study were 6 months of age at the project onset and were obtained from the Comparative Medicine Program of Indiana University School of Medicine and Purdue University (West Lafayette, IN). All pigs had normal estrous cycles at the initiation of the study and were sire-matched between treatments. The obese diet utilized in this study was comprised of a base pelleted pig feed (LabDiet Swine Diet 20% Fructose 5KA6, Purina Mills Inc.) supplemented (percentage by weight) with soybean oil (17.1%), cholesterol (2%), cholate (0.7%), corn oil (2.3%), and granular fructose (8.9%). The control diet fed in this study was the Rund diet (UIUC) comprised of corn (57.5%) and soy (40%) supplemented with vitamins and minerals. During the eight-month study period, control pigs (n = 9) were fed approximately 2200 kcal of the pelleted control diet per pig per day and obese pigs (n = 10) were fed approximately 4570 kcal of obese diet per pig per day. After a three-month diet induction period, pigs remained on their respective diets and were sampled for a five-month study period. Water was available *ad libitum*. Pigs were housed by dietary treatment group in pens of 2–4 pigs and were exposed to a 12 hour light:dark cycle. After eight months on their respective diets, pigs were euthanized when they had ovarian follicles of 9–10 mm in size on ultrasound. Euthanasia was achieved via administration of an overdose of sodium pentobarbital.

### Sample Collection

Blood collection and ovarian ultrasound were performed on each pig twice per week on fixed days during the five-month study. Blood was assessed for LH, FSH, progesterone (P4), estradiol (E2), dehydroepiandrosterone (DHEAS), androstenedione (A4), testosterone (T), fructosamine, insulin, leptin, glucose, and lipid profiles. Fructosamine was measured to assess long-term glucose homeostasis. Pigs were weighed and morphometric measurements taken once per week throughout the study period, including crown to rump length, thoracic girth, abdominal girth, and height. Pigs were trained to the use of a Panepinto low stress sling restraint system for purposes of ovarian ultrasound [27–29]. Pigs were checked daily for estrus by application of back-pressure in the presence of a boar, which was used in conjunction with serum hormone and ovarian ultrasound data to determine cycle length.

### Assessment of glucose homeostasis

The Precision Xtra glucometer (Abbott Laboratories, Bedford, MA) was validated for assessment of blood by the simultaneous collection of nine serum and nine whole blood samples from each treatment group. The serum samples were processed at the University of Illinois, College of Veterinary Medicine Veterinary Diagnostic Lab (CVMVDL) and the whole blood samples were measured on the glucometer. The glucose concentrations determined from the serum and whole blood samples were significantly correlated (R = 0.92; *p*<0.001) (SAS 9.1.3, SAS Inc., Cary, NC). Fasting (12–16 hour fast) blood glucose was assessed twice weekly. To rule out the development of diabetes in obese pigs, fructosamine was measured every other month (University of Illinois at Urbana-Champaign CVMDL, Urbana, IL). Leptin and insulin serum concentrations were measured monthly with a multi-species and porcine-specific RIA, respectively (Linco/Millipore Corporation, Billerica, MA). The leptin RIA was run at an average binding of 38.1% (n = 2) and had an assay sensitivity of 930 pg. The insulin RIA was run at an average binding of 43.3% (n = 9) and had an assay sensitivity of 0.34 μU. Insulin resistance by modified HOMA-IR (homeostatic model assessment-insulin resistance) was calculated [18].

### Plasma lipids

Total plasma cholesterol (mg/dL), triglyceride (mg/dL), low-density lipoprotein (LDL; mg/dL), and high-density lipoprotein (HDL; mg/dL) concentrations were assayed every other month as previously described (Cholesterol EZ, Triglyceride EZ)[18].

### Intravenous Glucose Tolerance Test (IVGTT)

The week prior to euthanasia an IVGTT was performed on each pig. Only nine obese pigs had the IVGTT procedure, as one obese pig died due to a cardiac arrest prior to this test. Pigs were placed in a Panepinto low stress sling restraint system [28] and administered isoflurane gas (Butler Schein, Dublin, OH) by facemask. Once pigs were in an anesthetic plane, they were placed in dorsal recumbency and a 14 gauge, 15 cm J wire IV catheter was placed percutaneously into the right jugular vein (Cook Medical, Bloomington, IN). A single blood pressure measurement was taken during the course of the IVGTT using a tail cuff sphygmomanometer (Critikon Dinamap 8100 / 8100T NIBP Monitor) [29]. Pigs recovered from isoflurane anesthesia for two hours prior to initiation of the IVGTT to avoid isoflurane-induced inhibition of insulin action [29].

To perform the IVGTT, pigs were placed in the Panepinto sling and a baseline blood sample was obtained after which an IV bolus of 0.5 g glucose/kg was administered. Post-bolus blood samples were collected every 10 minutes for 60 minutes post-bolus for analysis of blood glucose (YSI 2300 Stat Plus analyzer, YSI, Yellowsprings, OH) and serum insulin concentrations. A modified HOMA-IR was calculated for each time point.

### Hormone radioimmunoassay

The following serum hormones were assessed bi-weekly throughout the five-month study period: E2, P4, A4, T, DHEAS, FSH, and LH. Assays for E2, P4, A4, T, FSH, and LH were validated for use with either swine serum or extracted samples as previously described [26]. To remove lipids from serum samples and steroids from binding proteins prior to radioimmunoassay (RIA) all steroid hormones, except DHEAS, were extracted using a diethyl ether and hexane-methanol procedure [30]. Extraction efficiency was monitored using tritiated hormones (~1000 cpm/1 ml serum), and was > 65% for all samples. All steroid hormones, except DHEAS, were assessed using Coat-A-Count kits (Siemens Medical Solutions Diagnostics). LH and FSH were assayed as previously described [26]. The sensitivity of the E2 assay was 4.5 pg, and this assay had an inter-assay precision of 2.0% and an intra-assay precision of 1.0%. The T assay sensitivity was 230 pg with an inter-assay precision of 5.0% and an intra-assay sensitivity of 2.1%. The sensitivity of the A4 assay was 360 pg and this assay had an inter-assay precision of 3.2% and an intra-assay precision of 0.9%. The P4 assay had a sensitivity of 50 pg and an inter-assay precision of 1.8% and an intra-assay precision of 1.8%. The LH assay had an assay sensitivity of 240 pg, and an inter-assay CV of 12.9% (n = 7) and an intra-assay CV of 5.3% (n = 7). The FSH had an inter-assay CV of 1.7% (n = 7) and the intra-assay CV of 4.2% (n = 7).

For assessment of A4 in theca cell culture media, the Coat-A-Count androstenedione kit was validated for cell culture medium. DHEAS was assessed using a Beckman Coulter double antibody kit (DHEA-S-7 RIA, DSL 2700, Beckman Coulter Inc, Brea, CA). Standards (25–2500 pg) were made by adding a known amount of DHEAS to PBS-1% gel and then making further dilutions in charcoal stripped pig serum. The DHEAS assay was run at an average binding of 26.8% (n = 7) and had an assay sensitivity of 1055 pg. The inter-assay precision was 5.0% and the intra-assay precision was 1.5%.

Follicular fluid from a subset of pigs (control, n = 4; obese, n = 6) also was analyzed. Fluid collected from bisected ovulatory sized (9–10 mm) follicles (8–13 follicles per pig) was assessed for the following steroid hormones: E2, P4, A4, T and 17-hydroxysteroid progesterone (17OHP). To remove lipids and proteins, follicular fluid (500 μl) was extracted with 3 ml of diethyl ether (Sigma Aldrich). Extraction efficiency was monitored as described previously and was > 90% for all follicular fluid extractions. Extracted samples were reconstituted in PBS-1% gel to the volume of the original follicular fluid sample (500 μl). All follicular fluid steroid hormone assays (Coat-A-Count, Siemens Medical Solutions Diagnostics) were validated for a buffer-based system. Samples for assessment of follicular fluid steroid hormones were diluted as follows: E2 (1:800 to 1:1600), 17OHP (1:20), A4 (1:33), T (1:17), and P4 (1:50).

### Follicular description

DVD videos of ovarian ultrasound were assessed on an LCD television (Vizio VO22LF 22-Inch 1080 pixel LCD HDTV, Vizio, Irvine, CA) using a DVD player with step-through capabilities (Toshiba SD 6100 with 1080 pixel upconversion, Toshiba, New York, NY). According to a previously published method [31], follicles on each ovary were counted and measured and a conversion factor determined the actual follicle size from the ultrasound image. Follicle size categories were: <3.5 mm, small; 3.5–6.5 mm, medium; 6.5–12.5 mm, ovulatory size; >12.5 mm, cyst [32].

### Ovarian histology

Pigs were euthanized and ovaries harvested when there were ovulatory size follicles (9–10 mm) present. One ovary from a subset of pigs (n = 3 from each treatment) was placed in Dietrich’s fixative (formalin, acetic acid, 95% ethanol, deionized water) for ovarian histology, whereas the other ovary was reserved for theca cell culture. Fixed ovaries were serially sectioned (5 μm thickness), stained with Mayer’s Haematoxalin (Sigma Aldrich) and Eosin Y Solution (Sigma Aldrich; H&E), and ovulatory sized follicles were examined at 100-400x for cell architecture.

### Primary theca cell culture

Primary theca cell cultures were established for a subset of pigs (control, n = 4; obese, n = 6). Ovulatory sized follicles (9–10 mm) were isolated and bisected. Theca interna and granulosa cell layers were stripped from the theca externa and connective tissue with forceps and granulosa cells mechanically removed from the theca interna. Theca interna tissue pieces were digested in a 15 ml conical tube with 0.1% collagenase XI (Sigma Aldrich) on a rotating rocker for 15–20 minutes. The theca cell pellet was resuspended in DMEM/F12 medium with phenol red supplemented with 15% charcoal stripped fetal bovine serum (FBS; Charcoal/Dextran Treated Fetal Calf Serum, Thermo Scientific Hyclone, Logan, UT), 1x vitamins (v/v; 100x Vitamins MEM modification, MP Biomedicals), and 1% penicillin-streptomycin-actinomycin (v/v; PSA, MP Biomedicals) and was passed through a 22 gauge needle to further dissociate individual cells prior to counting with 0.2% (v/v) trypan blue solution (Trypan Blue Solution 0.4%, Gibco, Invitrogen, Carlsbad, CA) using a hemocytometer. Theca cells were plated at a density of 250,000 live cells per well in 750 μl of the above-described cell culture medium in 24 well tissue-culture-treated Costar plates (Costar 3524, Costar, Cambridge, MA). Cells were cultured in humidified 95% air and 5% CO_2_ at 37°C and were allowed to reach a minimum of 60% confluence prior to treatment (48 hours).

Three wells per treatment were washed twice with the treatment medium before application of treatments (750 μl). Treatments included: control, LH (10 ng/ml, porcine, Sioux Biochemical Inc., Sioux Center, IA), insulin (100 ng/ml, porcine, Sigma Aldrich), and LH + insulin (10 ng/ml and 100 ng/ml). All treatment medium consisted of DMEM/F12 with 1% PSA, 1x vitamins and 1% SSS (v/v; Synthetic Serum Substitute, Irvine Scientific, Santa Ana, CA). After 48 hours, treatment medium was removed from each well and frozen at -20°C for steroid hormone analysis. Theca cells were trypsinized with 200 μl of 0.25% trypsin with EDTA (v/v 0.5% Trypsin EDTA, Invitrogen, Carlsbad, CA), counted in a 0.2% trypan blue solution using a hemocytometer, and frozen at -80°C in 300 μl β mercaptoethanol RLT lysis buffer (Qiagen Inc, Valencia, CA) for subsequent gene expression analysis.

### Quantitative real time PCR

Theca, and granulosa cells from a subset of pigs (control, n = 4; obese, n = 6) were assessed for gene transcript levels. Total RNA was extracted from cell aliquots of 100,000 cells using the RNeasy Mini Kit (Qiagen Inc) and an on-column DNase treatment (RNase-free DNase Set, Qiagen Inc). Total RNA was quantified (Nanodrop 3300, Thermo Scientific, Wilmington, DE) followed by bioanalysis (Agilent 2100 Bioanalyzer, Agilent Technologies, Inc., Santa Clara, CA) for RNA quantity and quality. RNA integrity scores fell within the following range: 6.2–9.1. To determine relative fold differences in transcript levels, a standardized amount of total RNA from each pig was reverse transcribed to cDNA (High Capacity cDNA Reverse Transcription Kit, Applied Biosystems Inc, Carlsbad, CA), diluted 1:5 with RNAse—free water, followed by quantitative real time PCR (qRT-PCR) (TaqMan Gene Expression Assay, Applied Biosystems Inc) with the same amount of cDNA per reaction on a 7900HT Fast Real-Time PCR System (Applied Biosystems Inc). The following target genes, responsible for the production of androgen compounds in the ovary, were assessed in theca interna: 3-beta-hydroxysteroid dehydrogenase 1 (*HSD3B1*), 17-beta-hydroxysteroid dehydrogenase 4 (*HSD17B4*), and 17-alpha-hydroxylase/17,20 lyase (*CYP17A1*). The following target genes were assessed in granulosa cells: 3-beta-hydroxysteroid dehydrogenase 1 (*HSD3B1*) and aromatase *(CYP19A1)*. Our endogenous gene, 18s ribosomal RNA (*RN18S1)*, was tested for tissues obtained from control and obese pigs and demonstrated similar replication amongst the treatment groups. We used the following probe/primer sets to perform qRT-PCR: Applied Biosystems TaqMan Gene Expression Assay for *HSD3B1*: Ss03391752_m1; Applied Biosystems TaqMan Gene Expression Assay for *HSD17B4*: Ss03394675_m1; Applied Biosystems TaqMan Gene Expression Assay for *CYP17A1*: Ss03394947_m1; Applied Biosystems TaqMan Gene Expression Assay for *CYP19A1*; Ss03384876_u1; Applied Biosystems TaqMan Gene Expression Assay for *RN18S1*: Hs99999901_s1.

### Statistical analysis

Metabolic data and reproductive data were analyzed as two separate data sets. Reproductive response variables included in the multivariate model were: LH, FSH, P4, E2, A4, T, DHEAS, small, medium, ovulatory, and cystic follicles. Two consecutive complete estrous cycles were analyzed per pig. Baseline estrous cycle data were divided into follicular and luteal phases. Follicular phase was defined as sample dates preceded by a decrease in P4 below 2 ng/ml and followed by an increase in P4 above 2 ng/ml, corresponding with the lack of a CL by ultrasound visualization. Luteal phase was defined as a period with P4 greater than 2 ng/ml with visualization of CL on ultrasound. Estrous cycle length was calculated from one point in time characterized by a nadir in P4 (ie, below 2 ng/ml) coupled with lack of CL on ultrasound and standing estrous behavior, to the next point in time demonstrating these three criteria. The area under the curve (AUC) was calculated for each treatment group for both glucose and insulin in the IVGTT procedure. For the individual IVGTT data of glucose, insulin and HOMA-IR, a repeated measures ANOVA analysis was utilized with a covariance structure of AH(1) (SAS 9.2, Inc.). Weekly weights and measures taken over the five months of diet treatment were averaged for all pigs and compared statistically. Normality of data was assessed using a Levene’s test of homogeneity and Shapiro-Wilk. Transformation of non-normal data was done logarithmically for quantitative data and using square root for count and ratio data. ANOVA analysis was done for follicular fluid hormones, and cell culture medium data with PROC MIXED using type 3 sums of squares with the PDIFF command to compare treatments (SAS 9.2, Inc.). For reproductive response variables, the ANOVA was run as repeated measures in time with a TOEP or AH(1) covariance matrix structure (SAS 9.2, Inc.). For all transformed, non-normal data, results from PROC MIXED were back transformed and the back-transformed data are shown. All data presented herein are least square mean ± SEM. The mRNA abundance of target genes was normalized to an endogenous control gene, *RN18S1*. The relative fold induction of each gene was then compared between obese and control gilts using the **Δ**CT method. In all statistical tests, *p*<0.05 was the criterion for statistical significance.

## Results

### Development of obesity and metabolic syndrome

Abdominal girth (control, 97.8 ± 3.6 cm; obese, 122.4 ± 3.6 cm; *p* = 0.0001), thoracic girth (control, 95.3 ± 3.3 cm; obese, 116.1 ± 3.0 cm; *p* = 0.0003), and weight (control, 57.8 ± 5.4 kg; obese, 91.1± 5.1 kg; *p* = 0.0002) were all greater in obese than control pigs. Crown rump length (control, 111.0 ± 2.3 cm; obese, 122.0 ± 2.0 cm; *p* = 0.02) and height (control, 58.6 ± 1.1 cm; obese, 62.7 ± 1.1 cm; *p* = 0.02) also were significantly greater in the obese as opposed to control pigs. Abdominal girth is directly proportional to increased stores of visceral adipose tissue in Ossabaw pigs as seen on computerized tomography scans [18].

Obese pigs had fasting hyperglycemia compared with control pigs (control, 63.9 ± 1.5 mg/dL; obese, 80.9 ± 5.1 mg/dL; *p<*0.0001). However, there was no difference in fasting insulin concentrations between obese and control pigs (control, 14.5 ± 1.5 μU/ml; obese, 15.0 ± 1.4 μU/ml; *p* = 0.8). Basal HOMA-IR scores also were similar between obese and control pigs (control, 4.3 ± 1.4; obese, 5.9 ± 1.4; *p* = 0.15). Despite being hyperglycemic, obese pigs were not diabetic as demonstrated by fructosamine levels, which were similar to control (control, 254.2 ± 8.2 μmol/L; obese, 254.8 ± 7.9 μmol/L; *p* = 0.95). Leptin concentrations were significantly higher in obese compared to control pigs (control, 6.0 ±1.0 ng/ml; obese, 16.0 ± 1.0 ng/ml; *p*<0.0001).

Obese pigs had elevated total cholesterol (control, 163.5 ± 44.2 mg/dL; obese, 985.6 ± 41.9 mg/dL; *p*<0.0001), LDL (control, 28.7 ± 42.77 mg/dL; obese, 808.7 ± 40.5 mg/dL; *p*<0.0001), HDL (control, 112.0 ± 4.3 mg/dL; obese, 148.8 ± 4.1 mg/dL; *p*<0.0001), and triglycerides (control, 65.4 ± 11.1 mg/dL; obese, 99.6 ± 10.5 mg/dL; *p* = 0.0007) as compared with control pigs. Additionally, the LDL:HDL ratio was significantly increased in obese as compared with control pigs (control, 0.4 ± 0.3; obese, 5.8 ± 0.3; *p*<0.0001). Obese pigs were hypertensive compared with control pigs (mean arterial pressure: control, 103.0 ± 1.5 mmHg; obese, 121.5 ± 1.1 mmHg; *p*<0.0001).

**Fig 1:**
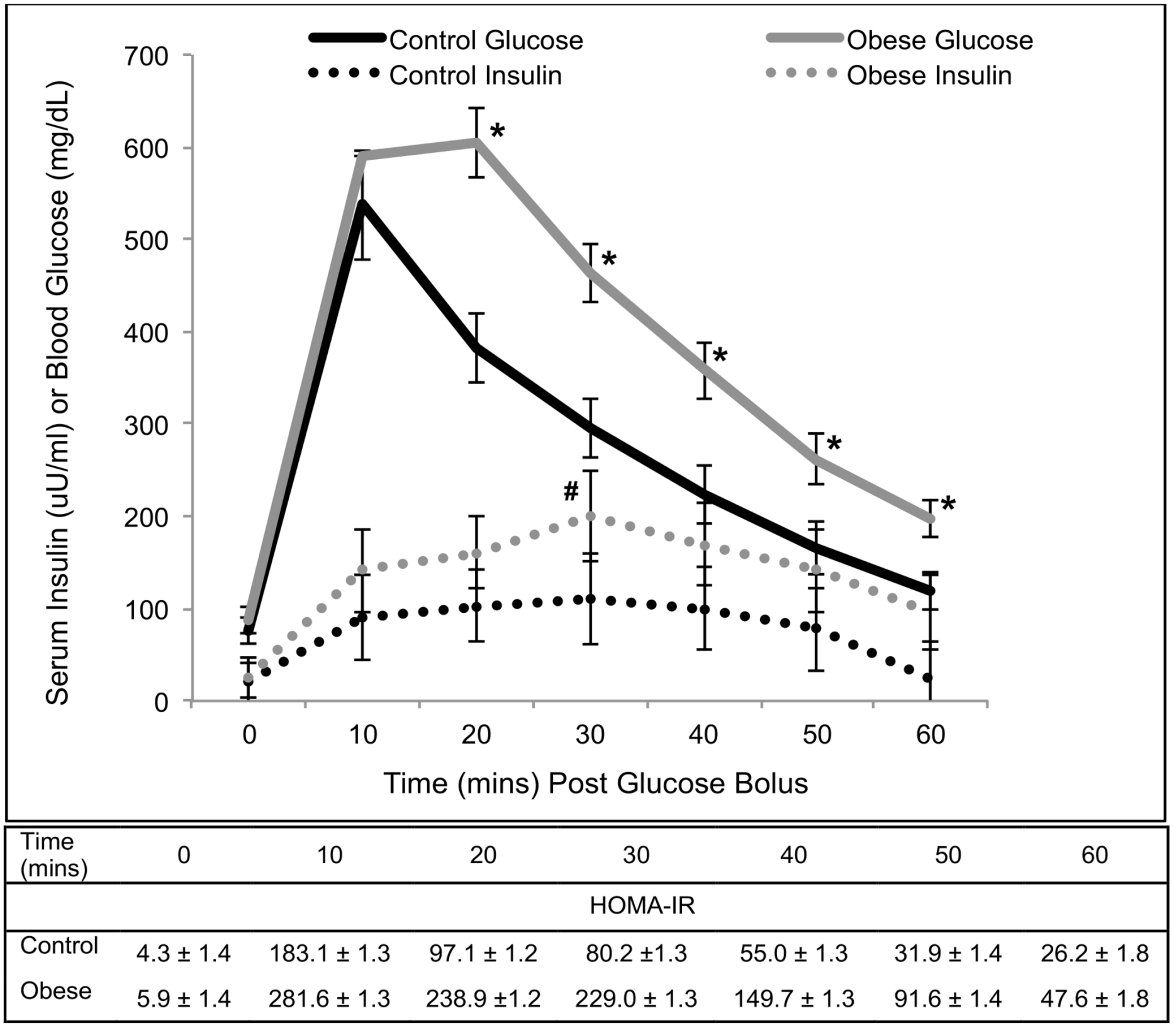
Intravenous glucose tolerance test in control (n = 9) and obese (n = 9) Ossabaw pigs. Points on the obese pig glucose curve marked with an * are significantly greater than corresponding points on the control pig glucose curve (p<0.01). The insulin point on the obese pig curve marked with a # is significantly greater than the corresponding point on the control pig curve (p = 0.05). Obese pigs had greater HOMA-IR values at all post-glucose bolus points as compared with control pigs (p≤0.05).

Although obese pigs had higher blood glucose concentrations compared to control pigs at all time points except for baseline and 10 minutes post glucose bolus, serum insulin was only greater in obese pigs compared with control pigs at 30 minutes post glucose bolus (Fig 1). Despite these findings, HOMA-IR was higher in obese pigs compared to control pigs at all time points post-glucose bolus (Fig 1). The IVGTT AUC for glucose was 17,204 mg/min/dL for control pigs and 27,783 mg/min/dL for obese pigs. The IVGTT AUC for insulin was 6,018 μU/min/ml for control pigs and 12,162 μU/min/ml for obese pigs. Therefore, based on the results of the glucose tolerance test, obese pigs were glucose intolerant and insulin resistant.

### The impact of obesity and metabolic syndrome on reproductive parameters

Obese pigs had a longer average estrous cycle length than control pigs (obese, 23.3 ± 1.5 days; control, 19.1 ± 0.1 days; *p* = 0.0001). Obese pig serum A4 concentration was significantly higher than control pig serum A4 concentration in the luteal phase only (Table 1). Serum T was not different between the two treatment groups (Table 1). Obese pigs had higher DHEAS concentrations during both the follicular and luteal phases of the estrous cycle (Table 1). Control pigs had higher serum LH concentrations than obese pigs, whereas obese pigs had higher serum FSH concentrations than control pigs (Table 1). Obese pigs had significantly higher serum P4 concentrations during the luteal phase only. There was no difference in serum E2 concentration between the two treatment groups (Table 1).

**Table 1:** Estrous cycle hormones in control (n = 9) and obese (n = 10) Ossabaw pigs.

Hormone	Control		Obese	
	Follicular	Luteal	Follicular	Luteal
Androstenedione (ng/ml)	0.22±0.04	0.28±0.03*	0.29±0.04	0.35±0.03*
Testosterone (ng/ml)	0.13±0.02	0.11±0.02	0.09±0.02	0.10±0.02
DHEAS (ng/ml)	0.17±0.01^#^	0.19±0.01*	0.23±0.01^#^	0.25±0.01*
Estradiol (pg/ml)	18.3±1.8	7.8±1.0	14.0±1.8	7.8±0.8
Progesterone (ng/ml)	1.3±2.0	11.0±0.9*	1.4±1.9	16.1±0.8*
FSH (ng/ml)	1.7±0.1^#^	2.2±0.1*	2.2±0.1^#^	2.6±0.1*
LH (ng/ml)	5.2±0.6^#^	3.2±0.4*	3.3±0.6^#^	2.2±0.1*

^#^ Significant difference between the control and obese pigs at the *p* <0.05 level during the follicular phase of the estrous cycle.

* Significant difference between the control and obese pigs at the *p* <0.05 level during the luteal phase of the estrous cycle.

**Fig 2:**
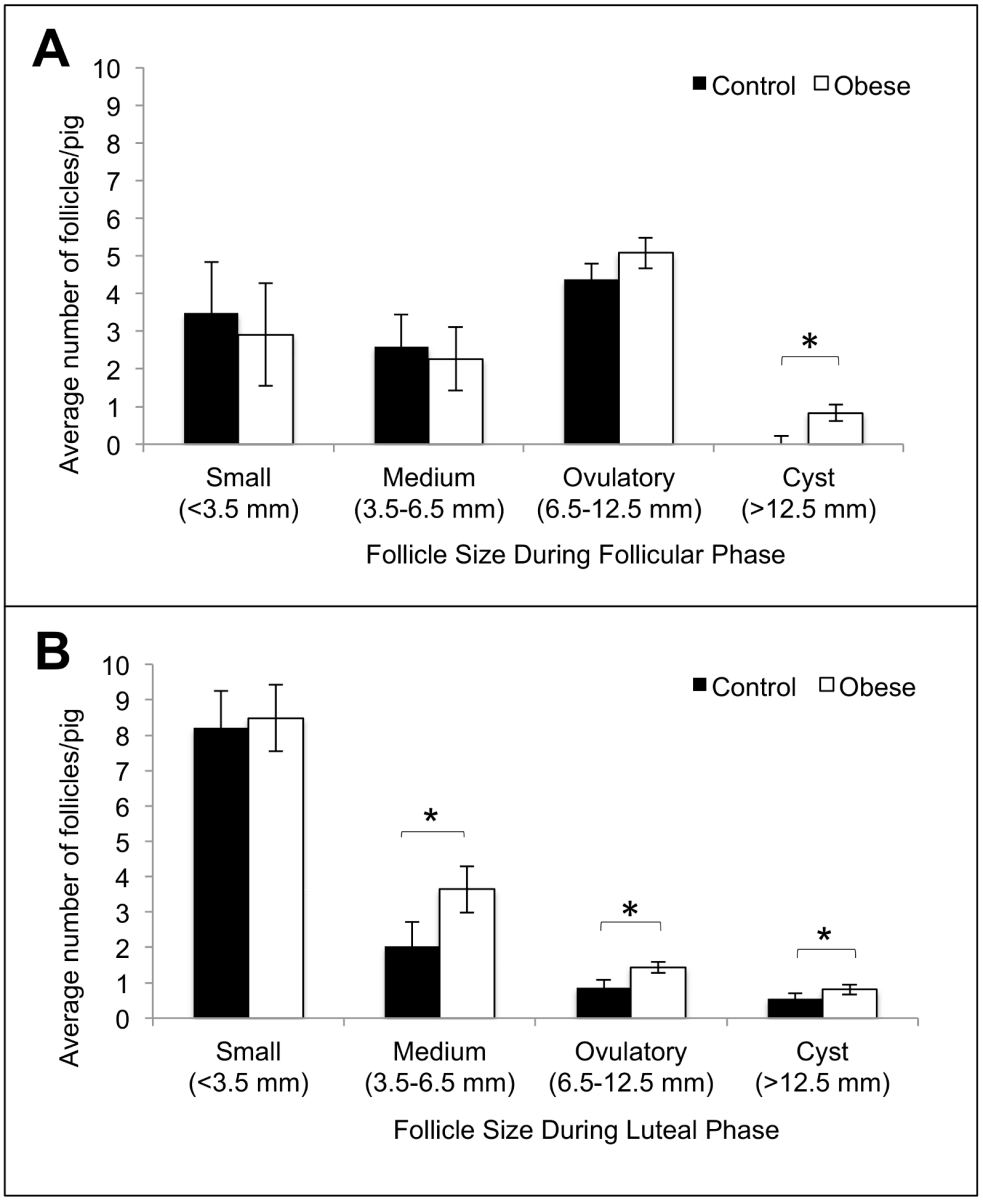
Average number of follicles per pig in follicular (A) and luteal (B) phases of the estrous cycle in control (n = 9) and obese (n = 10) Ossabaw pigs. Within a given follicle category, significant differences between control and obese pigs are marked with an * (*p*<0.05).

Obese pigs had significantly more cystic follicles (>12.5 mm) than control pigs in both the follicular and luteal phases of the estrous cycle (Fig 2A and 2B). Cystic follicles persisted on the ovaries of control pigs for 3.0 ± 1.4 days and on the ovaries of obese gilts for 3.8 ± 2.6 days. Cysts were found under several circumstances: as the only structures on the ovary, with other ovulatory size follicles, and with luteal tissue. There were no cysts present on the ovaries from pigs from either treatment group at the time of euthanasia and tissue harvest. Obese pigs had significantly more medium (3.5–6.5 mm) and ovulatory size (6.5–12.5mm) follicles than control pigs during the luteal phase of the estrous cycle only (Fig 2B). All ovaries from control pigs had normal theca interna and granulosa cell layers in all large follicles visualized within the ovarian cortex as determined by histology. One of the three obese pigs had hypertrophied theca interna and granulosa cell layers in two out of four ovulatory size follicles visualized within the ovarian cortex as determined by histology.

**Fig 3:**
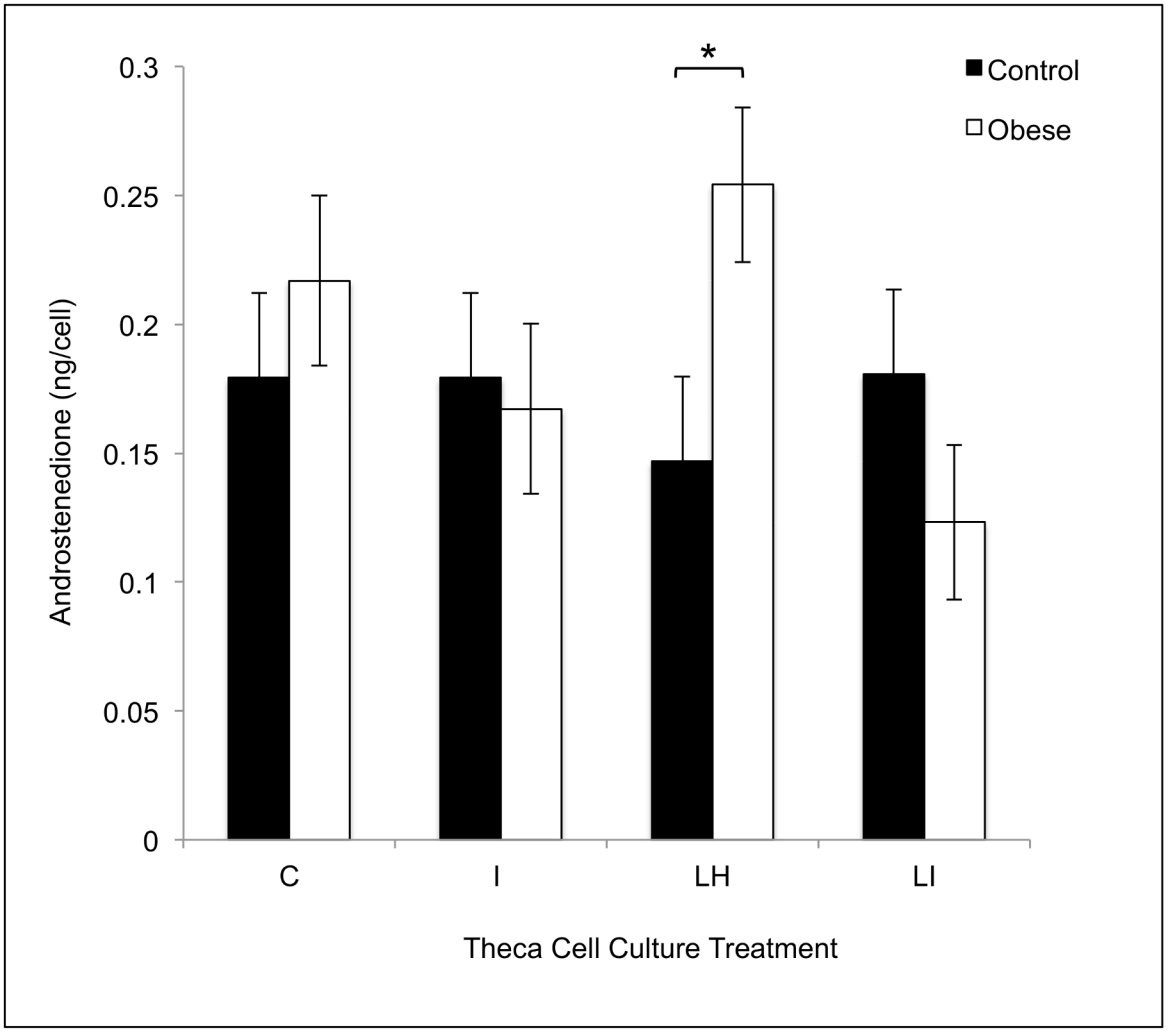
Theca cell androstenedione production in response to control (C), LH (LH; 10 ng/ml), insulin (I; 100 ng/ml) or LH + insulin (LI; 10 ng/ml + 100 ng/ml) treatments in vitro in control (n = 4) and obese (n = 6) pigs. Within a given cell culture treatment, significant differences between control and obese Ossabaw pigs are marked with an * (*p*<0.05).

**Fig 4:**
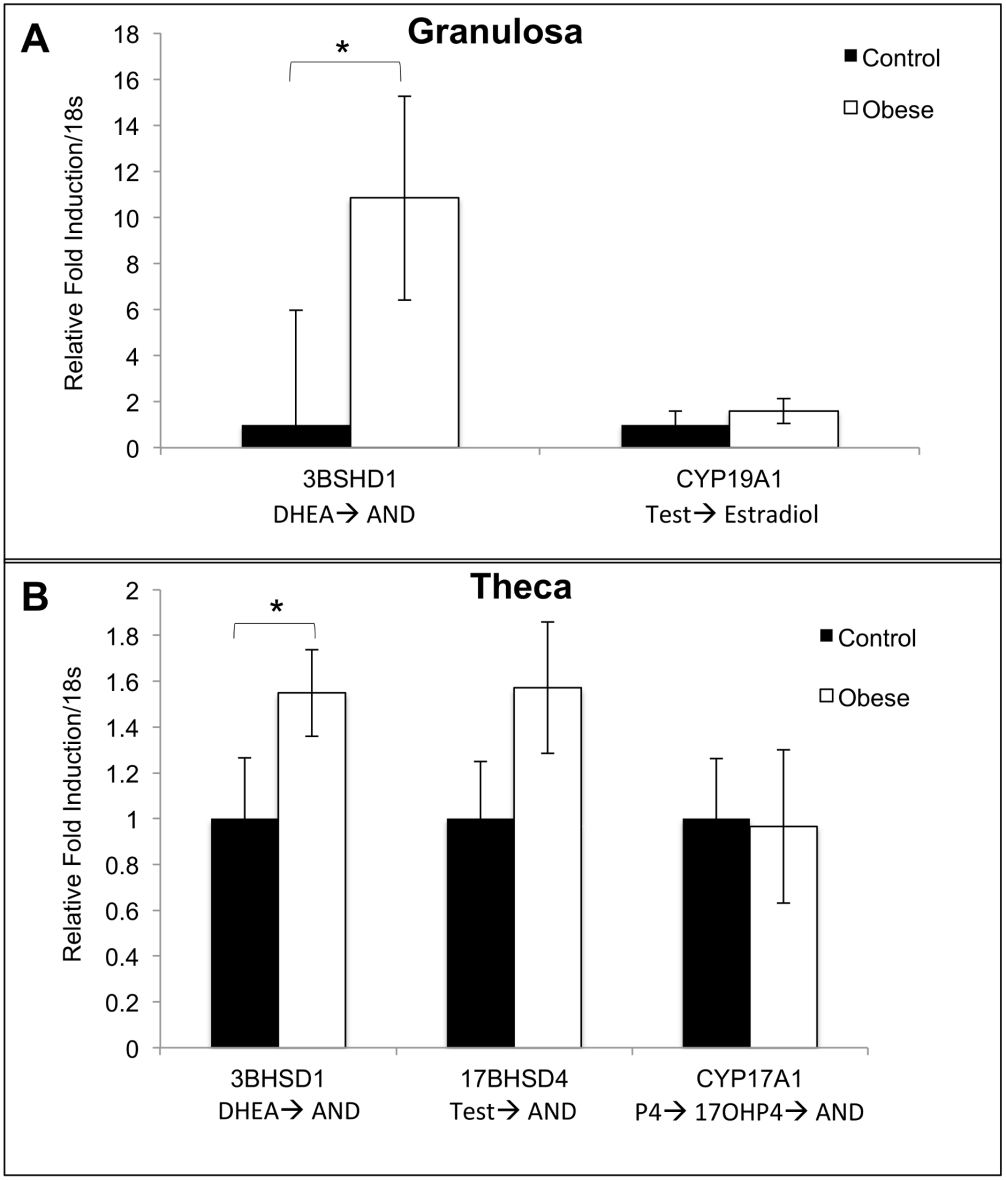
Naïve granulosa and theca cell steroidogenic enzyme gene expression in control (n = 4) and obese (n = 6) Ossabaw pigs. Cells were collected immediately following euthanasia and follicle collection. Within a given gene, differences in relative fold induction/18s between control and obese pigs are shown with an * (*p*<0.05). P4 = progesterone; DHEA = dehydroepiandrostereone; Δ4 = androstenedione; 17OHP4 = 17-alpha-hydroxy-progesterone; Test = testosterone; E2 = estradiol.

**Fig 5:**
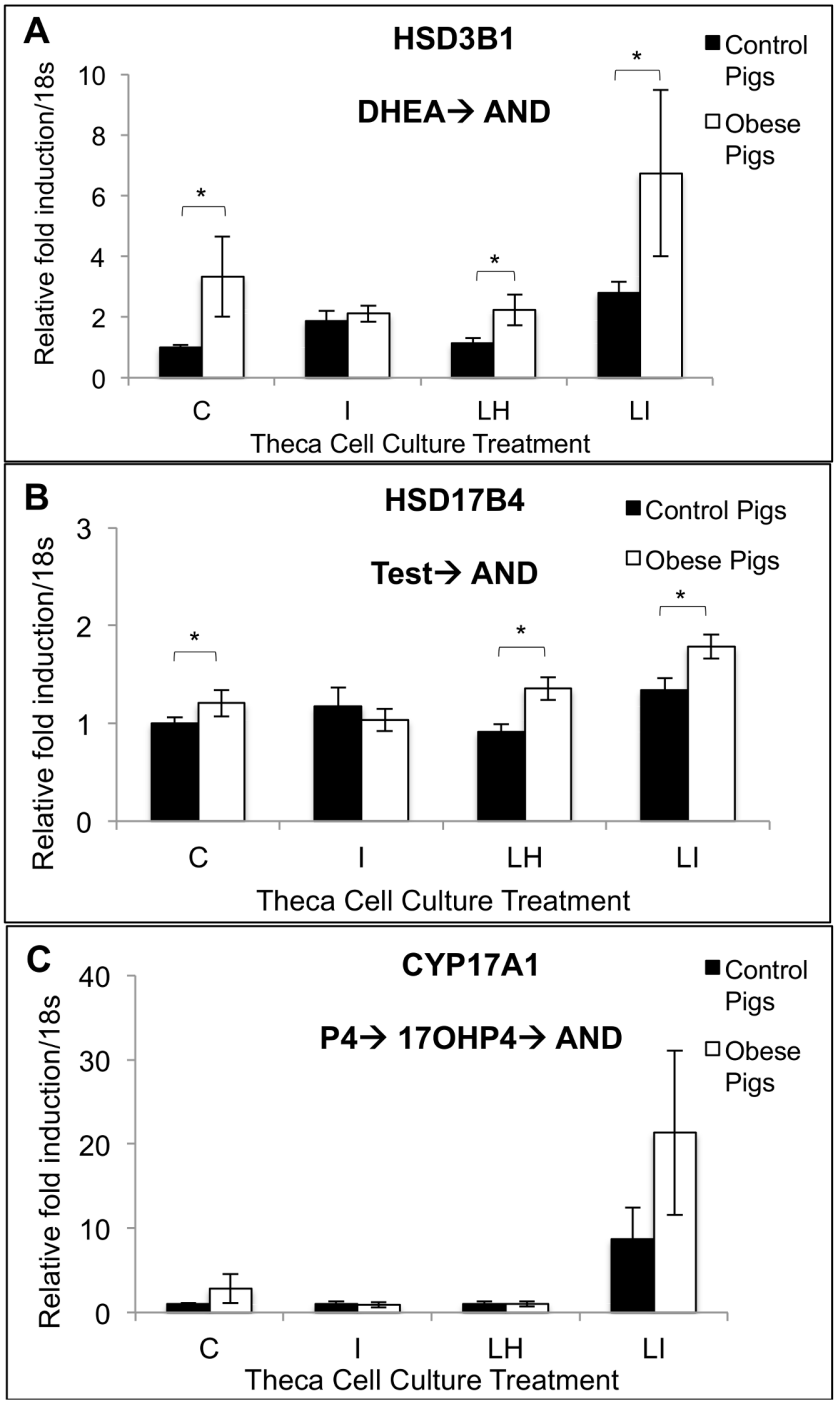
Theca cell gene expression of *HSD3B1* (A), *HSD17B4* (B) and *CYP17A1* (C) in response to control, LH (10 ng/ml), insulin (100 ng/ml) or LH + insulin (10 ng/ml + 100 ng/ml) treatments in vitro in control (n = 4) and obese (n = 6) pigs. Within a given cell culture treatment and gene, significant differences in relative fold induction/18s between control and obese Ossabaw pigs are marked with an * (*p*<0.05). P4 = progesterone; DHEA = dehydroepiandrostereone; AND = androstenedione; 17OHP4 = 17-alpha-hydroxyprogesterone; T = testosterone.

There was no difference in the absolute concentrations of any follicular fluid steroid hormone in control and obese pigs (A4: control: 50.0 ± 43.6 ng/ml; obese: 97.9 ± 39 ng/ml; T: control: 18.5 ± 9.5 ng/ml; obese: 23.7 ± 8.5 ng/ml; E2: control: 72.8 ± 25.2 ng/ml; obese: 31.3 ± 22.5 ng/ml; P4: control: 35.9 ± 17.8 ng/ml; obese: 60.7 ± 16.0 ng/ml; 17-α-hydroxyprogesterone: control: 0.6 ± 0.3 ng/ml; obese: 0.4 ± 0.3 ng/ml). Theca cells from obese pigs produced increased amounts of A4 in cell culture in response to treatment with LH (10 ng/ml) as compared with control pigs (Fig 3). Granulosa and theca cells from obese pigs showed increased *HSD3B1* transcript levels compared to granulosa and theca cells from control pigs (Fig 4A and 4B). In cell culture, theca cells from obese pigs, as compared with control pigs, showed increased *HSD3B1* and *HSD17B4* transcript levels in control, LH, and LH + insulin wells (Fig 5A and 5B). There was no difference in CYP17A1 transcript levels between the treatment groups in this experiment (Fig 5C).

## Discussion

The major findings of this study are: 1) young Ossabaw mini-pigs fed a hypercaloric, high fat/cholesterol/fructose diet for a short term period (eight months) developed abdominal obesity and metabolic syndrome; 2) young, obese Ossabaw mini-pigs had extended estrous cycles, were hyperandrogenemic, and had increased numbers of medium, ovulatory and cystic follicles; 3) LH-treated theca cells in culture from young, obese Ossabaw mini-pigs had elevated A4 production, and control, LH, and LH + insulin-treated theca cells in culture from young, obese Ossabaw mini-pigs had up-regulated expression of *HSD3B1 and HSD17B4* genes; and 4) primary theca and granulosa cells from young, obese Ossabaw mini-pigs had up-regulated *HSD3B1* gene expression compared to control pigs.

We have previously demonstrated that older, female Ossabaw pigs (6–8 years old at study onset) fed a hypercaloric, high fat/cholesterol/fructose diet for a long-term period (13 months) develop abdominal obesity and metabolic syndrome [26]. However, the ability to induce obesity and metabolic syndrome in a shorter length of time in a young Ossabaw pig (6–14 months old) decreases the financial cost of this model, while maintaining its useful metabolic features. Furthermore, obese Ossabaw pigs show decreased phosphorylation of the AMPK α subunit in skeletal muscle [33]. Such evidence indicates that the development of obesity, insulin resistance, and metabolic syndrome in hypercaloric, high fat fed Ossabaw mini-pigs is multifactorial, involving the AMPK pathway as well as other unknown mechanisms. As the development of obesity and metabolic syndrome in humans is similarly multifactorial, the Ossabaw pig is well suited as a model animal for the study of obesity and metabolic syndrome in humans. The young, obese Ossabaw mini-pig demonstrates many of the reproductive pathologies seen in obese women including an extended cycle length, hyperandrogenism, decreased LH, and aberrant steroidogenesis, supporting the potential use of the obese Ossabaw mini-pig as a model in which to study the effects of obesity on ovarian dysfunction.

Obese Ossabaw mini-pigs in this study demonstrated an extended estrous cycle which approximates the prolonged menstrual cycle of obese women [34]. The normal estrous cycle in a pig ranges from 18–21 days. The cycle length of the control pigs was within this range, while estrous cycle length was protracted in the young, obese pigs. Abdominal fat is specifically associated with menstrual disorders in women [35]. Furthermore, glucose intolerance and the grade of obesity in women is correlated with the probability of oligo- or amenorrhea [34]. One possible mechanism for anovulation and amenorrhea in obese women is increased production of cortisol by the adrenal cortex [36] which is known to suppress hypothalamic GnRH release, thereby contributing to menstrual disorders [37]. In this study we found that obese Ossabaw pigs, as compared with lean control Ossabaw pigs, had elevated serum DHEAS concentrations. Although sulfotransferase 2A1, which is responsible for rendering DHEA into DHEAS, is also found in the liver, its highest concentrations are found in the adrenal cortex [38]. This finding indicates that young obese Ossabaw pigs may have abnormal steroidogenic function in the adrenal cortex, similar to obese humans, which may contribute to their extended cycle length.

Young obese Ossabaw pigs had increased numbers of follicles in the larger antral follicle size classes. Obesity is associated with decreased LH concentrations in women [7] some of whom may have an attenuated follicular response to exogenous gonadotropins such as those used in IVF cycles [39–43]. The increased numbers of cystic follicles seen in obese Ossabaw pigs may indicate an inappropriate response of the follicle to the LH surge. Although our serum LH data must be interpreted judiciously as samples were collected only twice weekly, we did find decreased serum LH concentrations in obese pigs. Interestingly, it has been shown in a species of bat (*Scotophilus heathii)* that increased adiposity and serum leptin concentrations are associated with abnormal folliculogenesis, decreased circulating LH concentrations, and decreased LH receptor levels on ovarian follicles [44], indicating that leptin may be involved in abnormal folliculogenesis and altered gonadotropin concentrations in obese animals, like the obese Ossabaw mini-pig.

Young obese Ossabaw pigs were hyperandrogenemic with respect to serum A4 and DHEAS only, suggesting aberrant steroidogenesis in the ovary and, possibly, the adrenal gland. Interestingly, obese Ossabaw pigs did not show increased serum T concentrations in ovulatory size ovarian follicles (9–10 mm). Pigs do synthesize ovarian T, with the greatest concentrations found in small antral follicles (<3.5 mm) [45]. Whereas the levels of steroid hormones in ovulatory size follicles from obese and lean Ossabaw pigs are similar to those found in other pig breeds [46], they are lower than those typically found in human ovulatory size follicles [47]. In addition to the ovary and adrenal gland, adipose tissue also generates androgens [48], with abdominal adipose tissue favoring the conversion of active androgens like T to weak androgens like A4 [49]. Given that A4 and DHEAS were significantly increased in obese pigs as compared with control pigs, it is possible the adrenal gland and adipose tissue depot may play a role in the hyperandrogenemic environment in this animal model. Another important point to consider is that pigs lack sex hormone binding globulin (SHBG) which is responsible for binding up to 98% of circulating T in women [50]. In swine, androgens bind weakly to albumin and, therefore, androgens are more biologically available to tissues.

Using in vitro experiments with theca interna cells, this study provides molecular evidence to support the steroidogenic findings from our previously published study in the Ossabaw pig [26]. The fact that theca interna cells from obese pigs produce increased concentrations of A4 in response to LH treatment in vitro indicates that obese pigs may have constitutively upregulated activity of theca interna steroidogenic enzyme activity of side chain cleavage enzyme (SCC), steroidogenic acute regulatory protein (StAR), 17-alpha-hydroxylase, 17, 20 lyase, 3-beta-hydroxysteroid dehydrogenase 1 (3βHSD1), and/or 17-beta-hydroxysteroid dehydrogenase 4 (17βHSD4). Gene expression analysis demonstrated that *HSD3B1* and *HSD17B4* were up-regulated in theca interna from obese pigs both in control wells as well as wells treated with LH and LH + insulin. These results suggest that 3βHSD1 and 17βHSD4 may be responsible for the increased production of A4 in theca interna cultures originating from obese pigs and treated with LH. Because naïve, non-cultured theca interna cells from obese pigs also had increased transcript levels of *HSD3B1*, and these pigs had elevated circulating A4 concentrations, this steroidogenic enzyme appears to be key in contributing to the androgenic milieu in obese Ossabaw pigs.

Insulin is known to contribute to androgen synthesis in theca interna through stimulation of the insulin receptor as well as direct stimulation of 17-alpha-hydroxylase, 17,20 lyase, and 3βHSD [51]. Furthermore, it is possible that theca cells from obese pigs with low circulating LH concentrations have up-regulated LH receptors on their theca cells, making them particularly sensitive to the stimulatory effects of LH on steroidogenesis. Although the transcript levels of steroidogenic enzymes HSD3B1 and HSD17B4 are increased in the theca interna of obese pigs in the control, LH, and LH + insulin treatments, the secretion of A4 is only greater following treatment with LH alone. Endogenous serum LH in the Ossabaw from this study was 2–5 ng/ml. Perhaps the theca interna had a large secretion of A4 following treatment with LH due to our choice of a supraphysiologic dose (10 ng/ml). This possibility is, however, somewhat unfounded by the fact that the obese Ossabaw had high endogenous serum androstenedione and that supraphysiologic doses would be more likely to result in LH receptor down-regulation. Although the obese gilts were not hyperinsulinemic, it is possible they have either sub-clinical insulin receptor insensitivity or down-regulation of insulin receptors in tissues, including the ovary. Administration of insulin at robust concentrations such as used in our in vitro experiment (ie, 100 ng/ml) could result in further down-regulation of insulin receptors and a lack of adequate response to the insulin treatment in culture. This pathophysiology may explain the drop in A4 secretion seen in the theca cells from obese pigs in the LH + insulin treated wells.

Another possible explanation for several of the reproductive pathologies seen in the obese Ossabaw pig, including increased A4 production and up-regulation of *HSD3B1* in both theca and granulosa cells, is that Ossabaw pigs are homozygous wild type for insulin like growth factor 2 (IGF2) [52]. In pigs, mice and humans, *IGF2* is a paternally linked gene and in pigs a polymorphism of *IGF2*-intron3-G3072A SNP [53] confers leanness. Therefore, as Ossabaw pigs lack the *IGF2* polymorphism they will deposit more adipose tissue than pigs with the *IGF2* polymorphism. Theca interna synthesizes small amounts of IGF2 that stimulates androgen and P4 production, causes granulosa cell proliferation, increased sensitivity of granulosa cells to LH, and up-regulation of P4 production in granulosa cells [54, 55]. In humans, an *IGF2* polymorphism causes the insulin resistance and hyperandrogenism in PCOS patients [56]. It is possible that the combination of the AMPKγ3 mutation and the wild type *IGF2* gene, which confers increased fat accumulation in pigs, provides a genetic foundation upon which the addition of a hypercaloric, high fat diet causes obesity and reproductive dysfunction in the Ossabaw pig. Furthermore, obese Ossabaw have a decreased conception rate (number of pregnancies/breeding attempts) and decreased litter size compared with control Ossabaw (unpublished data). Future work in this model animal will focus on the assessment of protein levels of steroidogenic enzymes in theca and granulosa cells and on the assessment of steroidogenic enzyme activity. Additionally, determination of the roles of insulin, LH, IGF2, and AMPK on defects in follicular and steroidogenic function in obese Ossabaw pigs will better characterize the potential benefits of this animal model.

This study as well as previous studies [18, 20–22, 57] have demonstrated that Ossabaw mini-pigs when fed a hypercaloric, high fat/cholesterol/fructose diet develop android obesity and all other components of metabolic syndrome, including hypertension, glucose intolerance, insulin resistance, and dyslipidemia. In older, obese Ossabaw pigs (6–8 years old) fed for a longer time period (13 months), obese pigs were hyperinsulinemic as well as hyperglycemic [26]. However, the dyslipidemia of obese pigs in this study was more severe than that seen in our previous study in older Ossabaw pigs. Reproductively, serum T was not different between control and obese pigs, irrespective of age of the animal [26].

## Conclusion

In summary, this model of younger Ossabaw pigs fed a high fat/cholesterol/fructose diet for a short length of time mimics many features of metabolic and reproductive dysfunction observed in obese women, including hyperglycemia, visceral adiposity, dyslipidemia, hypertension, protracted estrous cycle length, hyperandrogenemia, development of ovarian cysts, and aberrant theca and granulosa cell steroidogenesis. Obese Ossabaw pigs have increased transcript levels and evidence for aberrant function for steroidogenic enzymes in the delta 4 steroidogenic pathway of the ovary, which may account for their hyperandrogenemia. As the development of obesity and metabolic syndrome is multifactorial, the young Ossabaw pig is well-suited as a model animal in which to study the effects of obesity and metabolic syndrome on ovarian function. Future research in this animal model will focus on the molecular mechanisms underlying obesity-induced ovarian dysfunction. Ultimately, the Ossabaw mini-pig may be a useful animal model in which to develop therapies to improve fertility in obese and/or hyperandrogenemic females or to examine the effects of obesity on the maternal-fetal environment and offspring health.

## References

[1] Flegal K, Carroll M, Ogden C, Curtin L. Prevalence and trends in obesity among US adults, 1999–2008. JAMA: The Journal of the American Medical Association. 2010;303(3):235–41. 10.1001/jama.2009.2014 20071471

[2] Eckel RH, Grundy SM, Zimmet PZ. The metabolic syndrome. The Lancet. 2005;365(9468):1415–28. 15836891 10.1016/S0140-6736(05)66378-7

[3] Chavarro JEMDS, Rich-Edwards JWMPHS, Rosner BAP, Willett WCMDD. Diet and Lifestyle in the Prevention of Ovulatory Disorder Infertility. Obstetrics & Gynecology. 2007;110(5):1050–8. 17978119 10.1097/01.AOG.0000287293.25465.e1

[4] Boots C, Stephenson MD. Does obesity increase the risk of miscarriage in spontaneous conception: a systematic review. Seminars in reproductive medicine. 2011;29(6):507–13. 10.1055/s-0031-1293204 22161463

[5] Jungheim ES, Moley KH. Current knowledge of obesity's effects in the pre- and periconceptional periods and avenues for future research. American Journal of Obstetrics and Gynecology. 2010;203(6):525–30. 10.1016/j.ajog.2010.06.043 20739012 PMC3718032

[6] Rosenfield RL, Bordini B. Evidence that obesity and androgens have independent and opposing effects on gonadotropin production from puberty to maturity. Brain Research. 2010;1364:186–97. 10.1016/j.brainres.2010.08.088 20816944 PMC2992573

[7] Diamanti-Kandarakis E, Bergiele A. The influence of obesity on hyperandrogenism and infertility in the female. Obesity Reviews. 2001;2(4):231–8. 10.1046/j.1467-789X.2001.00041.x 12119994

[8] Norman RJ, Noakes M, Wu R, Davies MJ, Moran L, Wang JX. Improving reproductive performance in overweight/obese women with effective weight management. Human Reproduction Update. 2004;10(3):267–80. 10.1093/humupd/dmh018 15140873

[9] Rondanelli M, Perna S, Faliva M, Monteferrario F, Repaci E, Allieri F. Focus on metabolic and nutritional correlates of polycystic ovary syndrome and update on nutritional management of these critical phenomena. Archives of gynecology and obstetrics. 2014;290(6):1079–92. 10.1007/s00404-014-3433-z 25200687

[10] Sivalingam VN, Myers J, Nicholas S, Balen AH, Crosbie EJ. Metformin in reproductive health, pregnancy and gynaecological cancer: established and emerging indications. Human Reproduction Update. 2014;20(6):853–68. 10.1093/humupd/dmu037 25013215

[11] Moran L, Tsagareli V, Norman R, Noakes M. Diet and IVF pilot study: Short-term weight loss improves pregnancy rates in overweight/obese women undertaking IVF. Australian and New Zealand Journal of Obstetrics and Gynaecology. 2011;51(5):455–9. 10.1111/j.1479-828X.2011.01343.x 21806596

[12] Esinler I, Bozdag G, Yarali H. Impact of isolated obesity on ICSI outcome. Reproductive Biomedicine Online. 2008;17(4):583–7. WOS:000259829800020. 18854116 10.1016/s1472-6483(10)60249-0

[13] Varga O, Harangi M, Olsson IAS, Hansen AK. Contribution of animal models to the understanding of the metabolic syndrome: a systematic overview. Obesity Reviews. 2010;11(11):792–807. 10.1111/j.1467-789X.2009.00667.x 19845867

[14] Szukiewicz D, Uilenbroek JTJ. Polycystic ovary syndrome—Searching for an animal model. J Med. 1998;29(5–6):259–75. ISI:000082002400002. 10503163

[15] Eisner JR, Barnett MA, Dumesic DA, Abbott DH. Ovarian hyperandrogenism in adult female rhesus monkeys exposed to prenatal androgen excess. Fertility and Sterility. 2002;77(1):167–72. 11779609 10.1016/s0015-0282(01)02947-8

[16] Welsh MJ, Rogers CS, Stoltz DA, Meyerholz DK, Prather RS. Development of a porcine model of cystic fibrosis. Transactions of the American Clinical and Climatological Association. 2009;120:149–62. . 19768173 PMC2744522

[17] Ziecik AJ, Derecka K, Gawronska B, Stepien A, Bodek G. Nongonadal LH/hCG receptors in pig: functional importance and parallels to human. Semin Reprod Med. 2001;19(1):19–30. Epub 2001/06/08. . 11394200 10.1055/s-2001-13907

[18] Dyson MC, Alloosh M, Vuchetich JP, Mokelke EA, Sturek M. Components of metabolic syndrome and coronary artery disease in female ossabaw swine fed excess atherogenic diet. Comparative Med. 2006;56(1):35–45. ISI:000237340300006. 16521858

[19] Sturek M, Alloosh M, Wenzel J, Byrd JP, Edwards JM, Lloyd PG, et al Ossabaw island minature swine: cardiometabolic syndrome assessment In: Swindle MM, editor. Swine in the Laboratory: Surgery, Anesthesia, Imaging, and Experimental Techniques. Boca Raton: CRC Press; 2007 p. 397–402.

[20] Edwards JM, Neeb ZP, Alloosh MA, Long X, Bratz IN, Peller CR, et al Exercise training decreases store-operated Ca2+entry associated with metabolic syndrome and coronary atherosclerosis. Cardiovascular Research. 2010;85(3):631–40. 10.1093/cvr/cvp308 19744946 PMC2802199

[21] Neeb ZP, Edwards JM, Alloosh M, Long X, Mokelke EA, Sturek M. Metabolic syndrome and coronary artery disease in Ossabaw compared with Yucatan swine. Comparative Med. 2010;60:300–15. 20819380 PMC2930329

[22] Lee L, Alloosh M., Saxena R., Van Alstine W., Watkins B.A., Klaunig J. E., Sturek M., Chalasani N. Nutritional model of steatohepatitis and metabolic syndrome in the Ossabaw miniature swine. Wiley Subscription Services, Inc., A Wiley Company; 2009 p. 56–67. 10.1002/hep.22904 PMC3254146 19434740

[23] Etherton T, Kris-Etherton PM. Characterization of plasma lipoproteins in swine with different propensities for obesity. Lipids. 1980;15(10):823–9. 10.1007/bf02534372 7442472

[24] Etherton TD. Subcutaneous adipose tissue cellularity of swine with different propensities for adipose tissue growth. Growth. 1980;44(3):182–91. . 7429285

[25] Bellinger DA, Merricks EP, Nichols TC. Swine Models of Type 2 Diabetes Mellitus: Insulin Resistance, Glucose Tolerance, and Cardiovascular Complications. ILAR Journal. 2006;47(3):243–58. 10.1093/ilar.47.3.243 16804199

[26] Newell-Fugate AE, Taibl JN, Clark SG, Alloosh M, Sturek M, Krisher RL. The effect of diet induced obesity on metabolic parameters and reproductive function in the female Ossabaw mini-pig Comparative Med. 2014;61(1):44–9. 24512960 PMC3929218

[27] Research IfLA. Guide for the care and use of laboratory animals. Washington, D.C.: National Academy Press; 2010.

[28] Panepinto LM, Phillips RW, Norden S, Pryor PC, Cox R. A comfortable, minimum stress method of restraint for Yucatan miniature swine. Lab Anim Sci. 1983;33(1):95–7. . 6834782

[29] Otis CR, al. e. Hyperglycemia-induced insulin resistance in diabetic dyslipidemic Yucatan swine. Comparative Med. 2003;53(1):53–64. . 12625507

[30] Bahr JM, Wang SC, Huang MY, Calvo FO. Steroid concentrations in isolated theca and granulosa layers of preovulatory follicles during the ovulatory cycle of the domestic hen. Biology of Reproduction. 1983;29(2):326–34. 10.1095/biolreprod29.2.326 6640023

[31] Knox RV, Taibl J, Altmyer M, Breen S, Canaday D, Visconti A. Assessment of follicle population changes in sows from day of weaning and during estrus using real-time ultrasound. Society of Reproduction and Fertility Supplement 2009;66:199–200. 19848283

[32] Knox RV. Recruitment and selection of ovarian follicles for determination of ovulation rate in the pig. Domest Anim Endocrinol. 2005;29(2):385–97. 10.1016/j.domaniend.2005.02.025 ISI:000230796500015. 15998504

[33] Spurlock ME, Gabler NK. The Development of Porcine Models of Obesity and the Metabolic Syndrome. The Journal of Nutrition. 2008;138(2):397–402. 18203910 10.1093/jn/138.2.397

[34] Castillo-Martinez L, Lopez-Alvarenga JC, Villa AR, Gonzalez-Barranco J. Menstrual cycle length disorders in 18- to 40-y-old obese women. Nutrition (Burbank, Los Angeles County, Calif). 2003;19(4):317–20. 10.1016/s0899-9007(02)00998-x . 12679164

[35] Douchi T, Kuwahata R, Yamamoto S, Oki T, Yamasaki H, Nagata Y. Relationship of upper body obesity to menstrual disorders. Acta obstetricia et gynecologica Scandinavica. 2002;81(2):147–50. 10.1034/j.1600-0412.2002.810210.x . 11942905

[36] Lado-Abeal J, Rodriguez-Arnao J, Newell-Price JDC, Perry LA, Grossman AB, Besser GM, et al Menstrual Abnormalities in Women with Cushing’s Disease Are Correlated with Hypercortisolemia Rather Than Raised Circulating Androgen Levels. J Clin Endocrinol Metab. 1998;83(9):3083–8. 10.1210/jc.83.9.3083 9745407

[37] Dubey AK, Plant TM. A suppression of gonadotropin secretion by cortisol in castrated male rhesus monkeys (Macaca mulatta) mediated by the interruption of hypothalamic gonadotropin-releasing hormone release. Biology of Reproduction. 1985;33(2):423–31. 10.1095/biolreprod33.2.423 3929850

[38] Rainey WE, Carr BR, Sasano H, Suzuki T, Mason JI. Dissecting human adrenal androgen production. Trends in Endocrinology and Metabolism. 2002;13(6):234–9. 12128283 10.1016/s1043-2760(02)00609-4

[39] Zhang D, Zhu Y, Gao H, Zhou B, Zhang R, Wang T, et al Overweight and obesity negatively affect the outcomes of ovarian stimulation and in vitro fertilisation: a cohort study of 2628 Chinese women. Gynecological endocrinology: the official journal of the International Society of Gynecological Endocrinology. 2010;26(5):325–32. 10.3109/09513591003632100 . 20192898

[40] Dechaud H, Anahory T, Reyftmann L, Loup V, Hamamah S, Hedon B. Obesity does not adversely affect results in patients who are undergoing in vitro fertilization and embryo transfer. European journal of obstetrics, gynecology, and reproductive biology. 2006;127(1):88–93. 10.1016/j.ejogrb.2005.12.009 . 16417960

[41] van Swieten ECAM, van der Leeuw-Harmsen L, Badings EA, van der Linden PJQ. Obesity and Clomiphene Challenge Test as predictors of outcome of in vitro fertilization and intracytoplasmic sperm injection. Gynecologic and obstetric investigation. 2005;59(4):220–4. 10.1159/000084347 . 15753618

[42] Spandorfer SD, Kump L, Goldschlag D, Brodkin T, Davis OK, Rosenwaks Z. Obesity and in vitro fertilization: negative influences on outcome. The Journal of reproductive medicine. 2004;49(12):973–7. . 15656214

[43] Lindheim SR, Sauer MV, Carmina E, Chang PL, Zimmerman R, Lobo RA. Circulating leptin levels during ovulation induction: relation to adiposity and ovarian morphology. Fertility and Sterility. 2000;73(3):493–8. 10.1016/s0015-0282(99)00578-6 . 10689001

[44] Srivastava RK, Krishna A. Increased circulating leptin level inhibits folliculogenesis in vespertilionid bat, Scotophilus heathii. Molecular and Cellular Endocrinology. 2011;337(1–2):24–35. 10.1016/j.mce.2011.01.017 . 21277349

[45] Wiesak T, Hunter MG, Foxcroft GR. Differences in follicular morphology, steroidogenesis and oocyte maturation in naturally cyclic and PMSG/hCG-treated prepubertal gilts. Journal of Reproduction and Fertility. 1990;89(2):633–41. 10.1530/jrf.0.0890633 2401990

[46] Conley AJ, Howard HJ, Slanger WD, Ford JJ. Steroidogenesis in the preovulatory porcine follicle. Biology of Reproduction. 1994;51(4):655–61. 10.1095/biolreprod51.4.655 7819446

[47] McNatty KP, Smith DM, Makris A, Osathanondh R, Ryan KJ. The Microenvironment of the Human Antral Follicle: Interrelationships among the Steroid Levels in Antral Fluid, the Population of Granulosa Cells, and the Status of the Oocyte in Vivo and in Vitro. J Clin Endocrinol Metab. 1979;49(6):851–60. 10.1210/jcem-49-6-851 511976

[48] Blouin K, Veilleux A, Luu-The V, Tchernof A. Androgen metabolism in adipose tissue: Recent advances. Molecular and Cellular Endocrinology. 2009;301(1–2):97–103. 19022338 10.1016/j.mce.2008.10.035

[49] Blouin K, Richard C, Belanger C, Dupont P, Daris M, Laberge P, et al Local Androgen Inactivation in Abdominal Visceral Adipose Tissue. J Clin Endocrinol Metab. 2003;88(12):5944–50. 10.1210/jc.2003-030535 14671194

[50] Selby C. Sex hormone binding globulin: origin, function and clinical significance. Annals of clinical biochemistry. 1990;27 (Pt 6):532–41. . 2080856 10.1177/000456329002700603

[51] Young JM, McNeilly AS. Theca: the forgotten cell of the ovarian follicle. Reproduction (Cambridge, England). 2010;140(4):489–504. 10.1530/rep-10-0094 . 20628033

[52] Lloyd PG, Fang M, Brisbin IL, Andersson L, Sturek M. AMP kinase gene mutation is consistent with a thrifty phenotype (metabolic syndrome) in a population of feral swine. The FASEB Journal. 2006;20(4):A299.

[53] Van Laere A-S, Nguyen M, Braunschweig M, Nezer C, Collette C, Moreau L, et al A regulatory mutation in IGF2 causes a major QTL effect on muscle growth in the pig. Nature. 2003;425(6960):832–6. http://www.nature.com/nature/journal/v425/n6960/suppinfo/nature02064_S1.html. 14574411 10.1038/nature02064

[54] Cara JF. Insulin-like growth factors, insulin-like growth factor binding proteins and ovarian androgen production. Hormone research. 1994;42(1–2):49–54. 10.1159/000184145 . 7525445

[55] Silva JRV, Figueiredo JR, van den Hurk R. Involvement of growth hormone (GH) and insulin-like growth factor (IGF) system in ovarian folliculogenesis. Theriogenology. 2009;71(8):1193–208. 10.1016/j.theriogenology.2008.12.015 19193432

[56] San Millán JL, Cortón M, Villuendas G, Sancho J, Peral B, Escobar-Morreale HF. Association of the Polycystic Ovary Syndrome with Genomic Variants Related to Insulin Resistance, Type 2 Diabetes Mellitus, and Obesity. J Clin Endocrinol Metab. 2004;89(6):2640–6. 10.1210/jc.2003-031252 15181035

[57] Kreutz RP, Alloosh M, Mansour K, Neeb Z, Kreutz Y, Flockhart DA, et al Morbid obesity and metabolic syndrome in Ossabaw miniature swine are associated with increased platelet reactivity. Diabetes, metabolic syndrome and obesity: targets and therapy. 2011;4:99–105. . 21660293 10.2147/DMSO.S17105 PMC3107692

